# Weipixiao attenuate early angiogenesis in rats with gastric precancerous lesions

**DOI:** 10.1186/s12906-018-2309-3

**Published:** 2018-09-10

**Authors:** Jinhao Zeng, Ran Yan, Huafeng Pan, Fengming You, Tiantian Cai, Wei Liu, Chuan Zheng, Ziming Zhao, Daoyin Gong, Longhui Chen, Yi Zhang

**Affiliations:** 1 Chengdu University of Traditional Chinese Medicine, Chengdu, 610075 China; 2 Guangzhou University of Chinese Medicine, Guangzhou, 510405 China; 3 Guangdong Provincial Institute of Chinese Medicine, Guangzhou, 510095 China; 4 Guangzhou Institutes of Biomedicine and Health, Chinese Academy of Sciences, Guangzhou, 510530 China

**Keywords:** Gastric precancerous lesions, ERK signaling, VEGF, HIF-1α, Herbal medicine, Weipixiao

## Abstract

**Background:**

Angiogenesis is a pathobiological hallmark of gastric cancer. However, rare studies focus on angiogenesis in gastric precancerous lesions (GPL). Weipixiao (WPX), a Chinese herbal preparation, is proved clinically effective in treating GPL. Here, we evaluated WPX’s anti-angiogenic potential for GPL, and also investigated the possibility of its anti-angiogenic mechanisms.

**Methods:**

HPLC analysis was applied to screen the major chemical components of WPX. After modeling N-methyl-N′-nitro-N-nitrosoguanidine (MNNG)-induced GPL in male Sprague-Dawley rats, different doses of WPX were administrated orally for 10 weeks. Next, we performed histopathological examination using routine H&E staining and HID-AB-PAS staining. In parallel, we assessed angiogenesis revealed by microvessel density (MVD) using CD34 immunostaining, and subsequently observe microvessel ultrastructure in gastric mucosa under Transmission Electron Microscope. Finally, we detect expression of angiogenesis-associated markers VEGF and HIF-1α using immunohistochemistry. Moreover, mRNA expressions of ERK1, ERK2, Cylin D1 as well as HIF-1α in gastric mucosa were determined by quantitative real-time reverse transcription- polymerase chain reaction.

**Results:**

We observed the appearance of active angiogenesis in GPL rats, and demonstrated that WPX could reduce microvascular abnormalities and attenuate early angiogenesis in most of GPL specimens with a concomitant regression of most intestinal metaplasia (IM) and a portion of gastric epithelial dysplasia (GED). In parallel, WPX could suppress HIF-1α mRNA expression (*P* < 0.01) as well as protein expression (although without statistical significance), and could markedly inhibit VEGF protein expression in GPL rats. Mechanistically, WPX intervention, especially at low dose, caused a significant decrease in the ERK1 and Cylin D1 mRNA levels. However, WPX might probably have no regulatory effect on ERK2 amplification.

**Conclusions:**

WPX could attenuate early angiogenesis and temper microvascular abnormalities in GPL rats. This might be partly achieved by inhibiting on the angiogenesis-associated markers HIF-1α and VEGF, and on the ERK1/Cylin D1 aberrant activation.

**Electronic supplementary material:**

The online version of this article (10.1186/s12906-018-2309-3) contains supplementary material, which is available to authorized users.

## Background

Gastric precancerous lesions (GPL) are generally defined as intestinal metaplasia (IM) and gastric epithelial dysplasia (GED). A direct link has been sought between gastric cancer and high prevalence of GPL worldwide [1–3], especially in Asia. Thus, blocking of gastric precancerosis toward malignant transformation is crucial to reducing the gastric cancer incidence. Nowadays, endoscopic mucosal resection is clinically applied only to severe GED and definite intramucosal carcinoma, yet most cases of GPL are not amenable to the endoscopic resection [4]. Moreover, vitacoenzyme tablet, a chemopreventive agent used in China, has been reported to be beneficial in treating atrophic gastritis and metaplasia lesion [5, 6]. Its therapeutic effect against GPL, however, was not completely confirmed. Therefore, the pursuit to discover alternative therapies to treat GPL is of high concern. In China, early herbal intervention has proved to be effective in halting and even reversing the majority of GPL [7, 8].

Angiogenesis, the sprouting of new capillaries from pre-existing vessels, is a fundamental process accelerating gastric cancer progression. Considerable evidence has demonstrated that excessive angiogenesis is significantly correlated with enhanced migratory and invasive activity, and therefore with poor prognosis [9–11]. Hence, targeting angiogenesis has been a central focus in gastric cancer treatment [12]. Angiogenesis is generally resulted from hypoxia orchestrated by multiple transcriptional activators, aiming at restoring intratumoral O_2_ delivery to hypoxic regions, thereby sustaining tumor growth [13]. Hypoxia inducible factor-1α (HIF-1α) has been proved to be a key regulator of cellular adaptation to hypoxia involved in angiogenesis process, and the process is frequently accompanied by a concomitant aberrant activation of vascular endothelial growth factor (VEGF) [14], which is essential for vascular development and can induce proliferation, differentiation and apoptosis of endothelial cells. Extracellular signal-regulated kinase (ERK), which may serve as specific effectors of VEGF signaling, play a vital role in sprouting endothelial cells during vascular development [15]. Aberrant activation of ERK signaling is closely linked to the carcinogenesis and development of gastric cancer [16]. ERK1 has been reported to overexpressed in 52.98% (231/436) cases of human gastric tumor, and high level of ERK1 protein expression was significantly correlated with age, tumor location, depth of invasion, Lauren’s classification, lymph node metastasis and tumor node metastasis (TNM) stage [17]. ERK2 is similarly important for predicting the prognosis of gastric cancer. The positive occurrence of ERK2 mRNA expression is 64.0% (32/50) in tumor tissues from patients with gastric cancer, which is markedly higher than that in non-cancerous tissues showing 18.0% (9/50) [18]. Furthermore, ERK2 expression level is significantly increased from TNM stage II to stage IV, suggesting a closely relationship between elevated ERK2 level and tumor invasion and TNM stage [18]. Cyclin D1 is involved in G1-S point of cell cycle, and thus induces cell proliferation and migration coexisted with angiogenesis. Cyclin D1 is frequently over-expressed in a substantial proportion of gastric cancer, and its expression may be governed by the ERK signaling [19, 20]. Although studies reported in recent years have addressed the pro-angiogenic role of the molecules in gastric cancer, it is unclear what role the molecules may play in gastric precancerosis.

Weipixiao (WPX) is a Chinese herbal prescription consisting of six herbs including *Astragalus Membranaceus, Pseudostellaria Heterophylla, Atractylodis Macrocephalae, Curcuma zedoaria, Salvia Miltiorrhiza* and *Hedyotis Diffusa Willd*. WPX prescription has been widely used in clinical for more than 15 years, and it shows satisfactory effects against “non-progressive GPL”. In previous clinical trials, different teams demonstrated that WPX possesses excellent abilities at relieving the clinical symptoms, reducing the precursor lesions (through gastroscopic and pathohistological examination), as well as partially eradicating *Helicobacter pylori* in GPL patients, and shows no toxic or side effects [21–23]. Experimentally, some WPX individuals exhibited potential anti-angiogenesis activities in several solid tumors. Extracts from *Astragalus membranaceus*, a “Yi Qi Hua Yu” herb (function to tonify qi and activate blood) belonging to WPX, could reduce angiogenesis-related molecules vascular endothelial growth factor and cyclooxygenase-2 in ovarian tumor-bearing mice [24]. Another active compound of *Astragalus membranaceus*, named formononetin, has been reported to repress hypoxia-induced retinal angiogenesis via the HIF-1α/VEGF signaling pathway [25]. Essential oil from another member *Curcuma zedoaria*, a widely used “Hua Yu Tong Luo” herb (function to dissipate blood stasis and free the collateral vessels), presented the anti-angiogenic activity, which therefore contributed to suppressing melanoma growth and lung metastasis [26]. A recent study suggested that Danshensu, a major water-soluble compound from *Salvia miltiorrhiza*, could improve microcirculation and remodel tumor vasculature, thereby enhancing the radioresponse for Lewis lung carcinoma xenografts in mice [27]. The aforementioned researchers revealed the anti-angiogenesis properties of several herbs from WPX. However, the anti-angiogenic potential of WPX in GPL treating, and the possibility of its anti-angiogenic mechanisms still remain unclear.

In this study, we tested whether early angiogenesis existed in N-methyl-N′-nitro-N-nitrosoguanidine (MNNG)-induced GPL rats. Also, we determined whether WPX had the ability against hyper- angiogenesis. In parallel, we screened potential anti-GPL constituents of WPX. More importantly, the hypothesis we wished to test was that the anti-angiogenesis property of WPX was associated with its regulatory effects on the angiogenesis-associated markers HIF-1α and VEGF, and on the ERK/Cylin D1 signal transduction pathway.

## Methods

### Animals

Male Sprague-Dawley rats weighting 150–170 g were obtained from Experimental Animal Center of Sun Yat-sen University (certificate No. 0111909). Animals were housed in a specific pathogen-free animal room, kept under optimal condition at 23 ± 1 °C and 40–60% humidity with a 12 h light-dark cycle, and fed with standard rat chow. All procedures relating to animal care and the animal research protocols conformed to the guidelines for the Care and Use of Laboratory Animal, issued by the Ministry of Science and Technology of China. This experiment was conducted in Guangdong Provincial Institute of Traditional Chinese Medicine, and was presented to the institutional ethical review board for approval (Ethic No. GDPITCM111018).

### Drugs and reagents

WPX comprises the following components: Huangqi (*Astragalus Membranaceus*)30 g, Taizishen (*Pseudostellaria Heterophylla*)15 g, Baishu (*Atractylodis Macrocephalae*)15 g, Eshu (*Curcuma zedoaria*)10 g, Danshen (*Salvia Miltiorrhiza*)10 g and Baihuasheshecao (*Hedyotis Diffusa Willd*)30 g. The herbs were provided and authenticated by the First Affiliated Hosipital of Guangzhou University of Chinese Medicine. The medical herbs were boiled with distilled water, and concentrated into a mixture containing crude drugs 1.5 g/mL. MNNG was supplied by Tokyo Kabushiki Kaisha, Japan (No. ZG4T1-FP). CD34 antibody was abtained from R&D Systems, USA (lot ZDP0112111); VEGF antibody was supplied by Abcam, UK (lot GR-116031-1); HIF-1α antibody was purchased from Santa Cruz Biotechnology, USA (lot L1212). Maxima™ SYBR Green/Fluorescein qPCR Master Mix (2X) was supplied by Fermentas, USA.

### High performance liquid chromatography (HPLC) analysis

HPLC analysis was performed to screen the potential chemical constituents of WPX preparation. Condition optimization of fingerprint: JADE-PAK ODS-AQ column (250 × 4.6 mm, 5 μm) and Inertsil ODS-SP column (4.6 × 150 mm, 5 μm) were utilized, with acetonitrile 0.1% phosphoric acid solution and acetonitrile 0.4% phosphoric acid solution as the mobile phase respectively, under full wavelength detection. The following chromatographic analysis conditions were determined: the separation was determined on Inertsil ODS-SP column (4.6 × 150 mm, 5 μm) with a mobile phase of acetonitrile (solvent A) 0.4% phosphoric acid solution (solvent B). For HPLC analysis, a 10 μL sample was injected into the column and eluted at a flow rate of 1.0 ml/min under room temperature. The detective wavelength was 203 nm.

### Grouping, modeling and treatment

SD rats were randomly divided into six experimental groups: control group (*n* = 9), model group (*n* = 11), model + vitacoenzyme group (VIT, *n* = 9, 0.2 g/kg/d), model + high-dose WPX group (H-WPX, *n* = 9, 15 g/kg/d), model + medium-dose WPX group (M-WPX, *n* = 9, 7.5 g/kg/d), and model + low-dose WPX group (L-WPX, *n* = 9, 3.75 g/kg/d). Based on the literatures [8, 28, 29], the GPL rat model was set up with minor modifications. Briefly, All the rats, except for control rats, were allowed to drink MNNG solution (200 μg·ml^− 1^) ad libitum, and underwent hunger-satiety shift every other day. At the end of 15th week, 2 random rats in the model group were humanely terminated with sodium pentobarbital (140 mg/kg i.p.) and examined for IM/GED. At the beginning of 16th week, the treated rats were administered WPX or VIT by gastrogavage for 10 consecutive weeks, while the control and the model rats were given 2 mL distilled water by gastrogavage once daily.

### Pathological examination

Animals were humanely euthanized with sodium pentobarbital (140 mg/kg i.p.) after 12 h fasting, and the stomachs were removed immediately, incised along the greater curvature, and fixed in 10% neutralized formalin solution. Then, each sample was embedded in paraffin wax and serially sectioned at 3 μm thick. The sections were stained with hematoxylin and eosin (H-E staining), and with high-iron diamine-alcian blue-periodic acid Schiff (HID-AB-PAS staining). Gastric tissues were examined macroscopically to identify IM and GED lesions in rats.

### Evaluation of microvessel density

To evaluate microvessel density (MVD) in gastric mucosa, CD34 expression was determined using EnVision immunohistochemistry. Quantification of MVD was specified by Weidner et al. [30]. Briefly, area of highest angiogenesis (also called hot-spot) were identified under low-power magnification (40× and 100×), and stained microvessels in three random views of the ‘hot-spot’ area were counted under high-power magnification (200×). The mean value of the three 200× field counts was recorded as MVD for each case. Any brown staining endothelial cell or cell cluster that was clearly separated from adjacent microvessels or other connective tissue was considered a single countable microvessel.

### Microvessel ultrastructure

The gastric mucosa tissue were sliced into 1 mm^3^ pieces and fixed with 2.5% glutaraldehyde in phosphate buffer for 2.5 h, and then re-fixed in 1% osmium tetroxide in phosphate buffer for 2 h. The tissues were dehydrated in a graded series of ethanol solutions and then immersion in a mixture of acetone and epoxy resin twice (2:1 for 3 h in the first time, 1:2 for overnight in the second time). Finally, the tissues were embedded in epoxy resin-filled capsules and heated at 70 °C overnight, ultrathin sections (60–80 nm) were sliced with LKB microtome. The sections were viewed and photographed under a transmission electron microscope (JEOL 100C, JEOL, Tokyo, Japan).

### Levels of ERK1, ERK2, Cyclin D1 and HIF-1α by RT-qPCR

The mRNA levels of ERK1, ERK2, Cyclin D1 and HIF-1α were determined by quantitative real-time reverse transcription-polymerase chain reaction (RT-qPCR) method using Maxima™ SYBR Green/Fluorescein qPCR Master Mix (Fermentas, USA) via IQ™5 real-time PCR detection system (Bio-Rad, USA). The PCR primers used were as follows: ERK1 (GenBank accession no. NM_011952; 104 bp) forward, 5′-CGGATTGCTGACCCT-3′ and reverse 5′-GTGTAGCCCTTGGAGTT-3′; ERK2 (GenBank accession no. NM_053842; 113 bp) forward, 5′-CAACCTCCTGCTGAAC-3′ and reverse 5′-GCGTGGCTACATACTC-3′; Cyclin D1 (GenBank accession no. NM_171992; 191 bp) forward, 5′-GCAGAAGTGCGAAGAGG-3′ and reverse 5′-GGCGGATAGAGTTGTCAGT-3′; HIF-1α (GenBank accession no. NM_024359; 132 bp) forward, 5′-CAACTGCCACCACTGATG-3′ and reverse 5′-CACTGTATGCTGATGCCTTAG-3′; 18S (GenBank accession no. M11188; 204 bp) forward, 5′-TCAGCCACCCGAGATT-3′ and reverse 5′-GCTTATGACCCGCACTTA-3′. The level of 18 s mRNA transcript was used to normalize all reported gene expression levels, and the data were analyzed using 2^-△△Ct^ method.

### Expression of VEGF and HIF-1α by immunohistochemistry

Formalinfixed and paraffin-embedded gastric tissues were cut at 3 μm thick. The EnVision immunohistochemical technology was utilized, VEGF protein immunoreactivity was shown as brown color in the cytosolic and perinuclear regions of gastric epithelial cells. HIF-1α positive staining was brown or brown yellow and was detected predominantly in the cytoplasm and nucleus. To access the protein expression levels of VEGF and HIF-1α, three visual fields were randomly selected from each slice under light microscope (100×), and then images were acquired and analyzed by Image Pro Plus 6.0 software. Quantification of VEGF and HIF-1α levels was determined using mean of integrated optical density (IOD).

### Statistical analysis

Data were presented as mean ± standard deviation (SD). Statistical analysis was performed using IBM SPSS 19.0 software (SPSS, Chicago, IL). One-way analysis of variance (ANOVA) was applied to analyze the comparisons among multiple groups. The comparison between two groups was performed with SNK method for the homogeneous variances, while the variances were heterogeneous, Dunnett’s T3 method should be adopted. A *P* value of less than 0.05 was considered as significant.

## Results

### HPLC profile

**Fig. 1:**
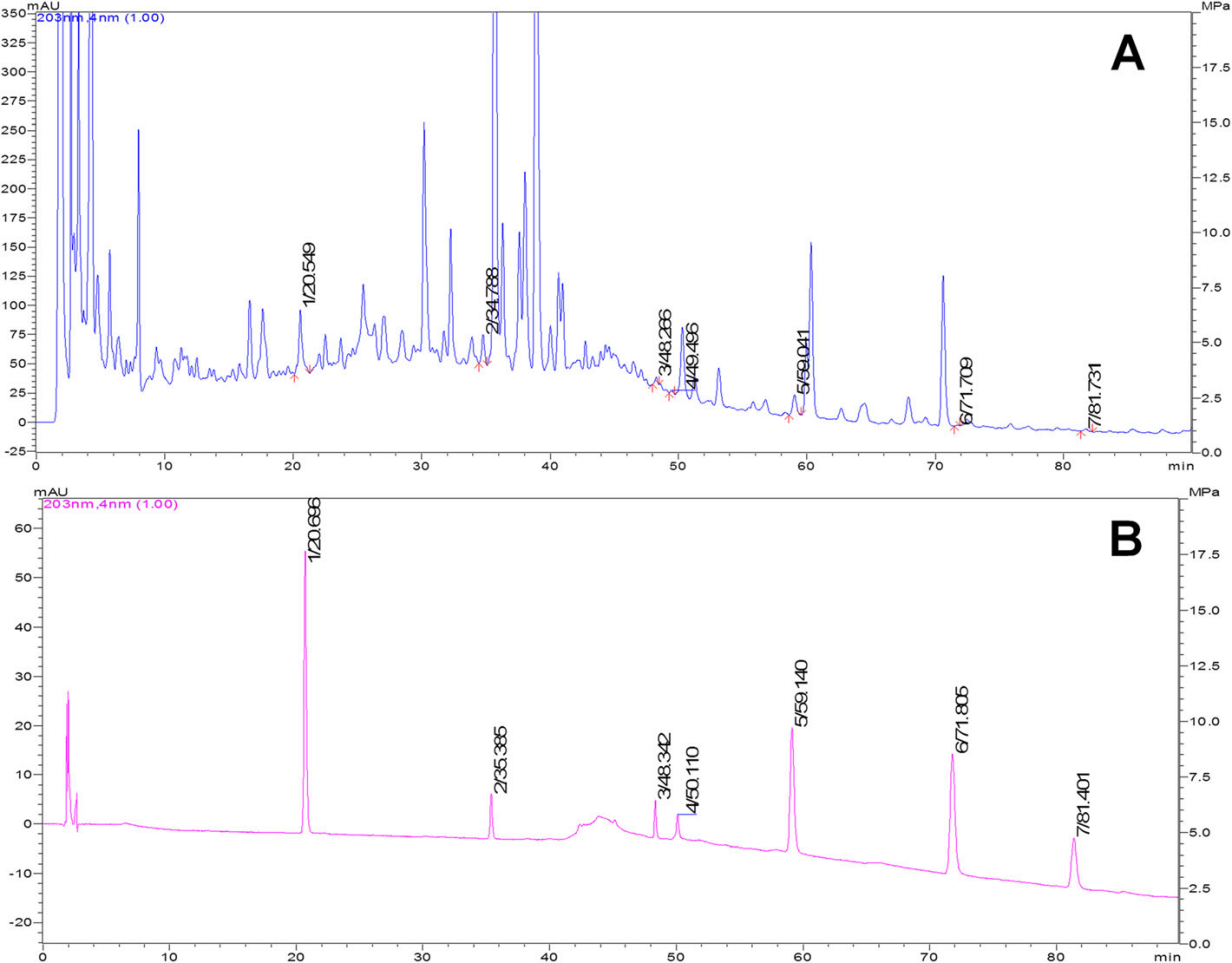
HPLC chromatogram of WPX test sample (**a**) and reference sample (**b**). Notes: Peak: 1, Calycosin-7-glucoside; 2, ginsenoside-Rg1; 3, ginsenoside-Rb1; 4, astragaloside IV; 5–7, atractylenolide III, II, and I, respectively

WPX possessed an excellent ability against GPL revealed by our previous clinical trials and animal testing, so we are curious about the major constituents of WPX polyherbal mixture. Figure 1 shows the HPLC chromatograms of WPX test sample (A) and reference sample (B). The retention times of the major chemical constituents were 20.5 min (Calycosin-7- glucoside), 34.8 min (ginsenoside-Rg1), 48.3 min (ginsenoside-Rb1), 49.5 min (astragaloside IV), 59.0 min (atractylenolide III), 71.7 min (atractylenolide II), and 81.7 min (atractylenolide I) (Fig. 1).

### WPX efficiently blocked and even reversed gastric intestinal metaplasia

**Fig. 2:**
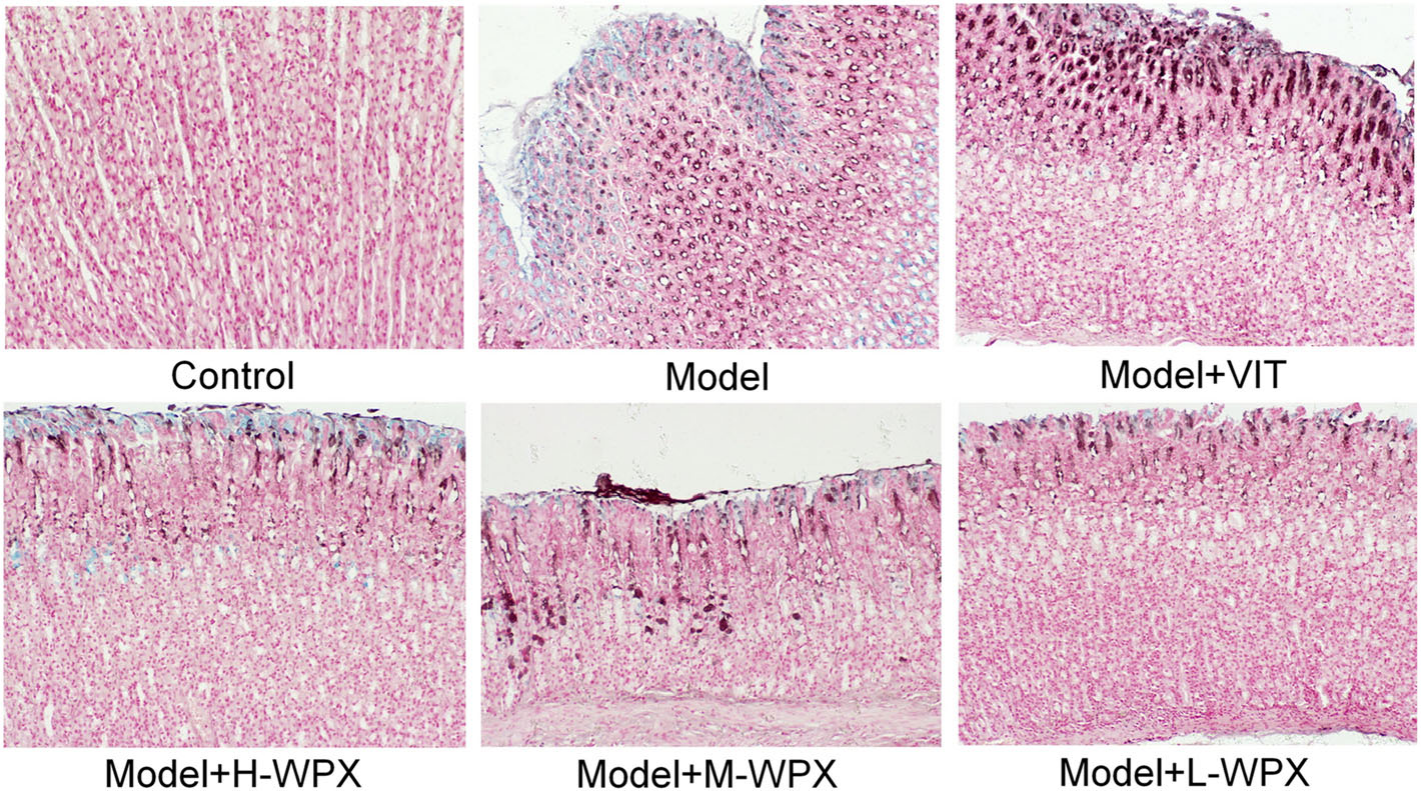
Histological evaluation of gastric intestinal metaplasia. Neutral mucins present in normal mucosa were stained red. Sialomucins expressed only in small intestinal-type metaplasia (S-IM) were stained blue, and sulfomucins present in colonic-type metaplasia (C-IM) were stained brown. Images of model gastric epithelium depicted prominent S-IM and C-IM lesions, which were dramatically reduced after WPX administration. *n* = 9 in each group. (HID-AB-PAS staining, 100×)

We evaluated the degree of IM lesion in gastric tissues by HID-AB-PAS staining. As depicted in Fig. 2, neutral mucins present in normal mucosa were stained red, gastric specimens from controls didn’t exhibit IM lesion. In model rats, sialomucins expressed only in small intestinal-type metaplasia (S-IM) were stained blue, and sulfomucins present in colonic-type metaplasia (C-IM) were stained brown, indicating that both S-IM and C-IM were widespread. In treated rats, IM lesion was regressed slightly in VIT-treated rats. Comparatively, IM lesion was regressed visibly in WPX-treated rats. Our observation revealed that WPX has a potent anti-IM capacity in GPL rats (Fig. 2).

### WPX partly ameliorated gastric epithelial dysplasia

**Fig. 3:**
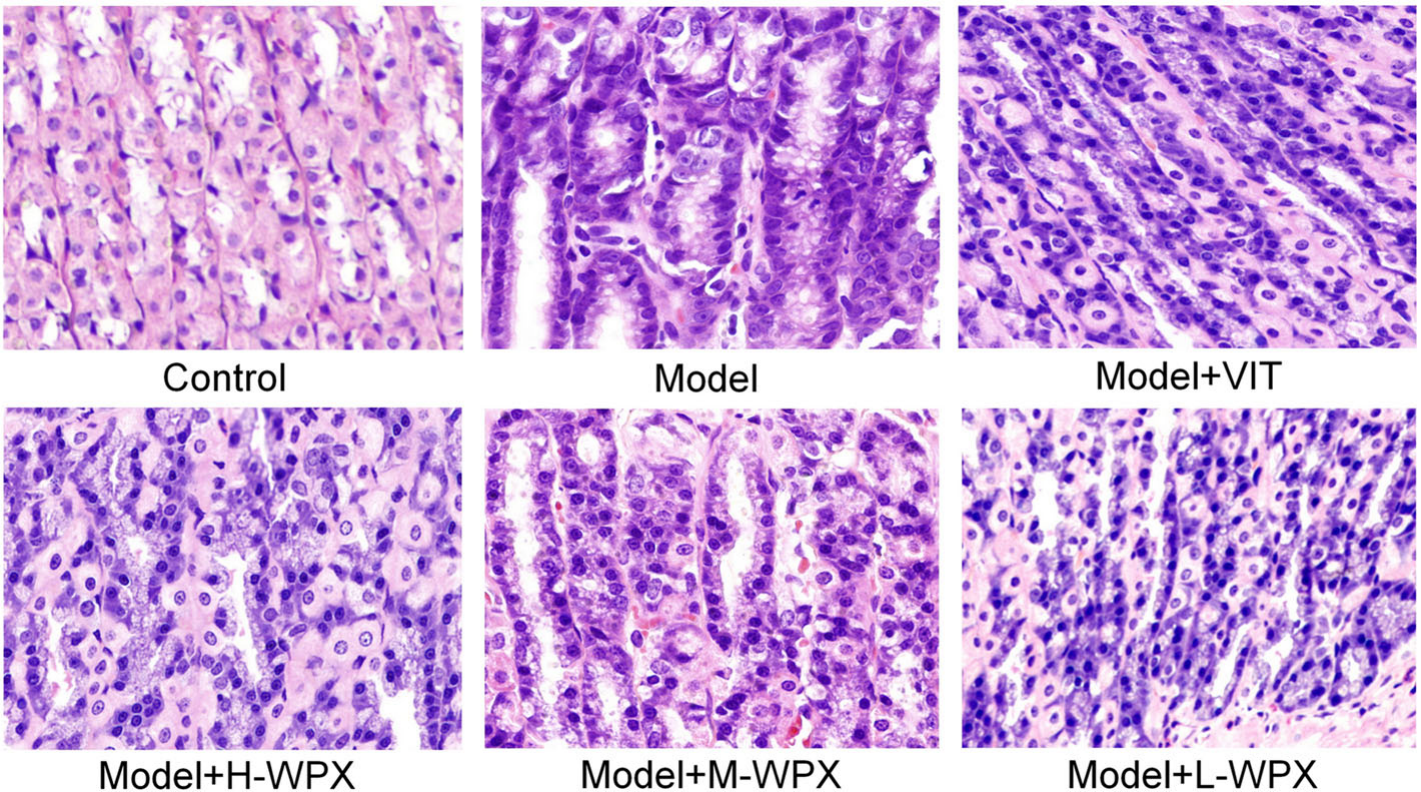
Histological evaluation of gastric epithelial dysplasia. Model gastric epithelium displayed GED pathology characterized by glandular architectural abnormalities such as splitting, elongated and crowded glands, back to back formation, as well as by cytological atypia with rounded, pleomorphic nuclei that display prominent nucleoli and loss of polarity. After WPX intervention, these GED pathological alterations, especially irregularities of glandular structure, were regressed in varying degrees. *n* = 9 in each group. (H&E staining, 100×)

To further investigate the anti-GPL effect of WPX, we also examined the GED lesion in H&E stained sections of gastric tissues. Histologically, the control gland and cell structure of gastric epithelium remained intact. By contrast, almost all model rats displayed GED pathology. In detail, gastric epithelium was characterized by architectural abnormalities showing splitting, elongated, crowded glands and back-to-back tubular structure, and also by cytological atypia with hyperchromatic nuclei, increased nuclear-cytoplasmic ratio, loss of nuclear polarity and occasional binucleation. Inflammatory infiltration was variable, and sometimes extensive. Occasionally, two model rats exhibited mild dysplasia, due to the multifocal nature of the dysplastic lesion. In most cases of WPX-treated rats, GED lesion alterations, especially tubular structure irregularities and inflammatory infiltration, were regressed in varying degrees. However, the treatment was not able to restore the GED pathology near to the normal tissues. In contrast, this GED-rescuing effect was not presented in most VIT-treated rats. These observations suggested that WPX could partly halt and even reverse dysplastic process, especially the “non-progressive GED” (Fig. 3).

### WPX reduced the CD34+ MVD level in GPL tissues

**Fig. 4:**
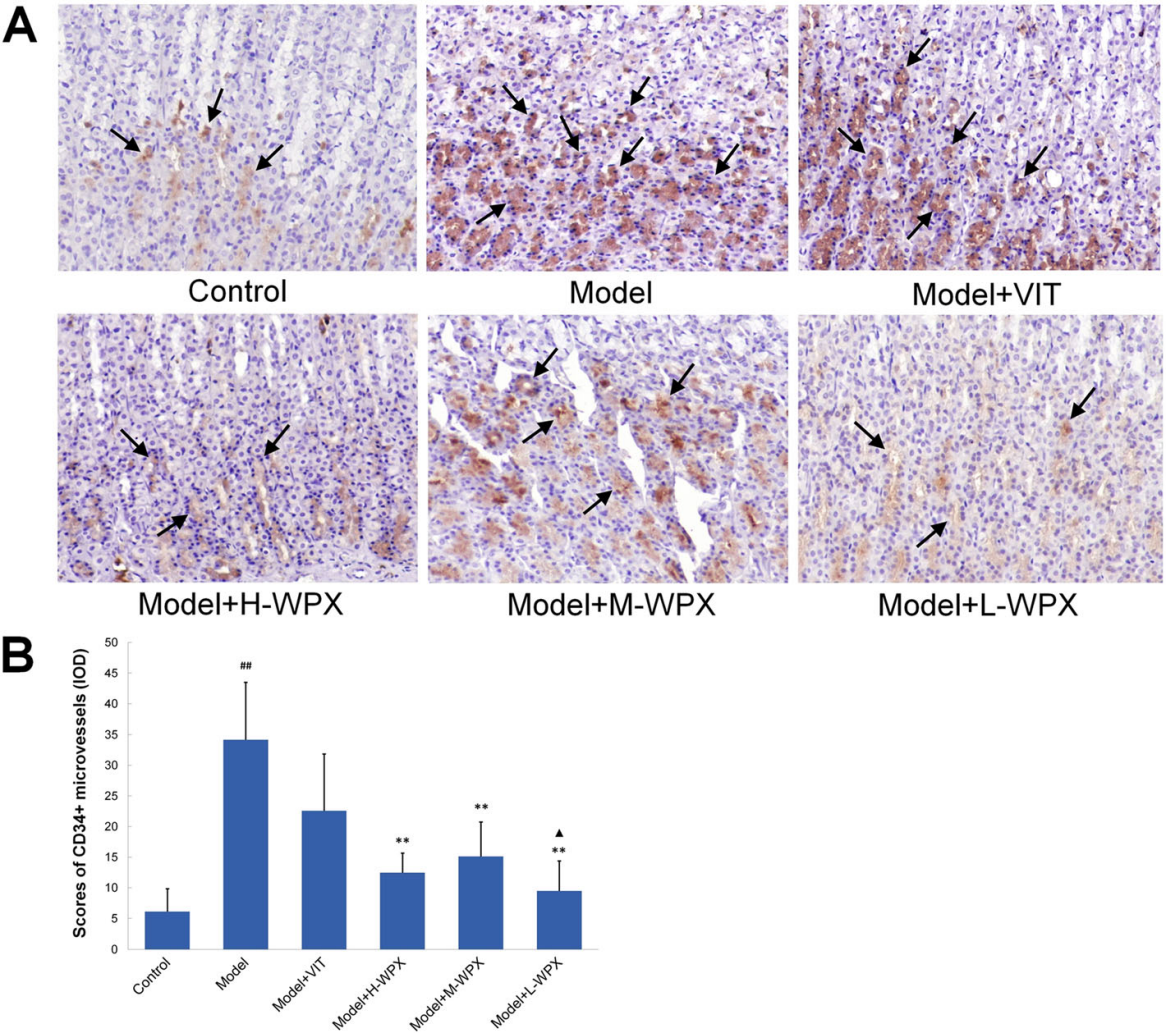
Evaluation of CD34-labelled microvessel density in gastric mucosa. **a** CD34-labelled microvessels in gastric mucosa from various groups. Some representative microvessels are indicated by black arrows. **b** Scores of CD34+ MVD levels. The results are expressed as mean ± SD (*n* = 9 in each group). Note: ^##^*P* < 0.01, vs Control; ^**^P < 0.01, vs Model; ^▲^*P* < 0.05, vs VIT. (IHC, 200×)

In order to identify whether early angiogenesis occur in GPL, we examined the angiogenic state in gastric tissues using CD34-labelled sections. As visualized by light microscopy, an increased number of CD34+ microvessels, which were suggestive of active angiogenesis, could be found in most cases of GPL tissues, whereas those in control tissues were minimal. Moreover, we found more GEDs with a higher number of microvessels than IMs, and more severe GEDs than mild or moderate GEDs. Occasionally, CD34+ microvessels were apparently abundant and distributed diffusely in two model rats all diagnosed with severe GED. By contrast, we observed a clear decreased CD34+ microvessel count in many cases of WPX-treated rats. Our data showed a statistic significant increase of CD34+ MVD level in model rats comparing to controls. But it dropped by at least half after WPX intervention, when in comparison to the non-treatment rats. Thus, WPX could effectively inhibit active angiogenesis in GPL rats (Fig. 4).

### WPX tempered microvascular abnormalities in GPL tissues

We further examined the morphological changes of microvessels in gastric mucosa under transmission electron microscope. In control rats, microvessels were clearly demarcated from the surrounding connective tissues. The microvessels appeared a normal vascular inner diameter, smooth basal lamina with uniform thickness, and also showed a complete and clear structure of basal lamina with homogeneous electron-density. Endothelial cell bordering the basal lamina was morphologically flat or elongated, with smooth and clear nuclear membrane and normal chromatin distribution.

In model rats, intervascular boundaries were ill-defined or invisible. Remodeled microvessels showed dilated vascular lumen but with a markedly decreased inner diameter, accompanied with clearly thickened, rough basal lamina which was often coated by abundant high-density granules aggregation. Some vascular lumens were partly or completely occluded by erythrocytes and neutrophils. Apart from these, features such as segmental breakup of basal lumina and increased vascular permeability also existed. Furthermore, endothelial cells displayed severe swelling and were shaped like grapes, with debased cytoplasm electron-density, nucleus chromatin condensation, as well as abundant pinocytotic vesicles. Abnormalitiesof vascular lumen, basal lamina and endothelial cell were still prominent in VIT-treated rats.

**Fig. 5:**
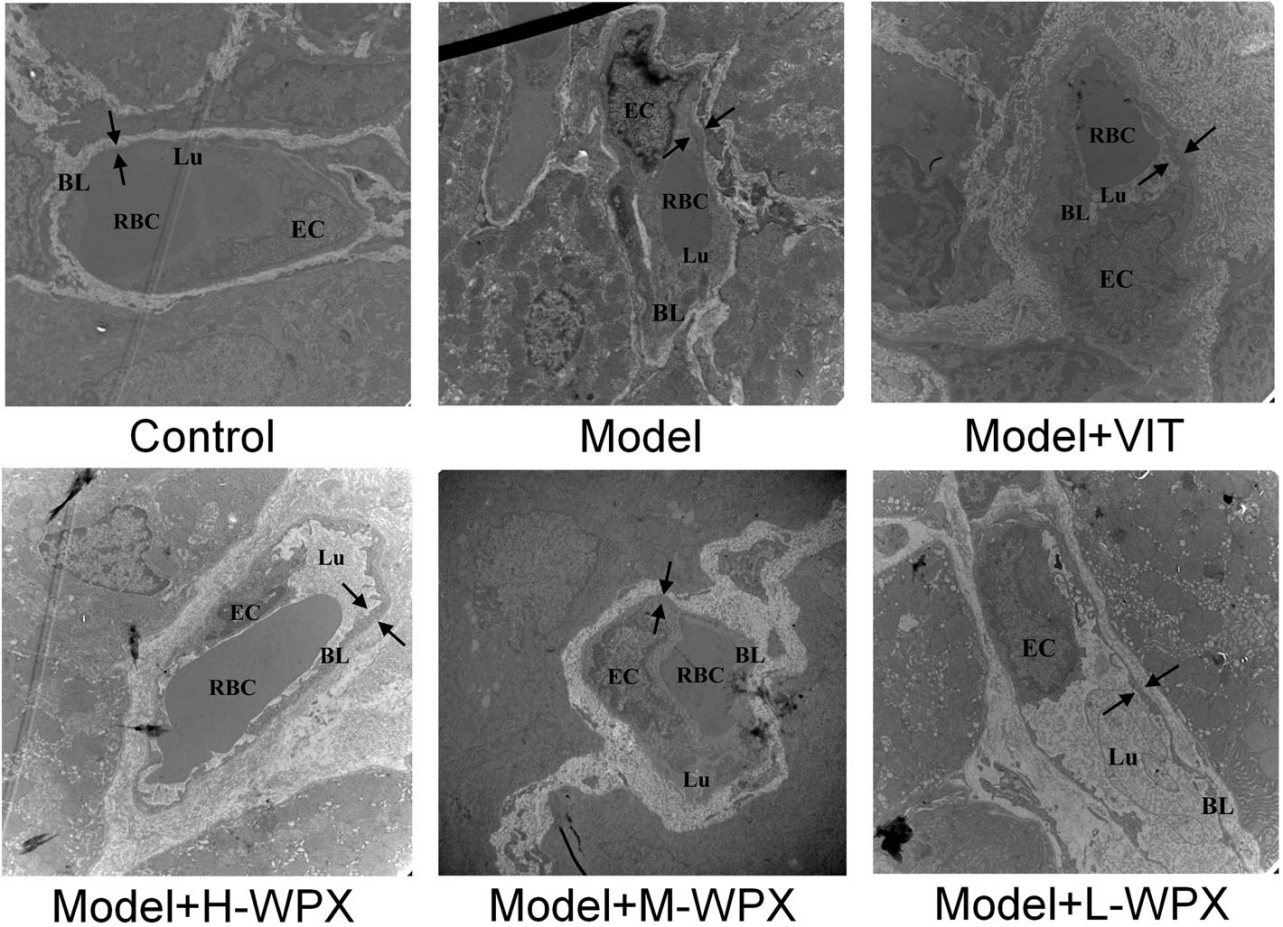
Representative electron micrographs of microvessels in gastric mucosa. **Control group**: TEM observation of control microvessel ultrastructures appeared intact, in terms of vascular lumen, basal lamina and endothelial cell. **Model group**: Microvessels lost their typical structures. Vascular lumen, frequently plugged by erythrocytes, was dilated but with a markedly decreased inner diameter. Clearly thickened, rough basal lamina was coated by abundant high-density granules aggregation. Segmental breakup of basal lumina and increased vascular permeability were also existed. Endothelial cells were conglobated and shaped as grapes, characterized by debased cytoplasm electron-density, nucleus chromatin condensation, as well as numerous pinocytotic vesicles. **Treatment group**: Microvascular abnormalities were still prominent in VIT-treated tissues. However, the abnormalities reversed markedly in WPX-treated tissues, especially in terms of vascular lumen and basal lamina. Even in a few cases, the microvessels were detected to ultrastructurally resemble the normal ones. Note: Opposing arrows mark the thickness of basal lamina; EC, endothelial cell; BL, basal lamina; Lu, lumen; RBC, red blood cell. *n* = 9 in each group. (10000×)

In WPX-treated rats, vascular lumen showed a mild-moderate decrease in inner diameter. Basal lamina coated by some high-density granule aggregation was still a little rough, and also with occasional breakup. Endothelial cells exhibited slight swelling and mild vacuolisation. Most of the nuclear membrane became clear and complete, and nucleus chromatin distribution also became normalized. Accordingly, WPX intervention could normalize ultrastructural alterations of vascular lumen, basal lamina and endothelial cell, thus showing the potent rescuing effect of WPX on microvascular abnormalities in GPL rats (Fig. 5).

### Effect of WPX on HIF-1α mRNA levels

**Fig. 6:**
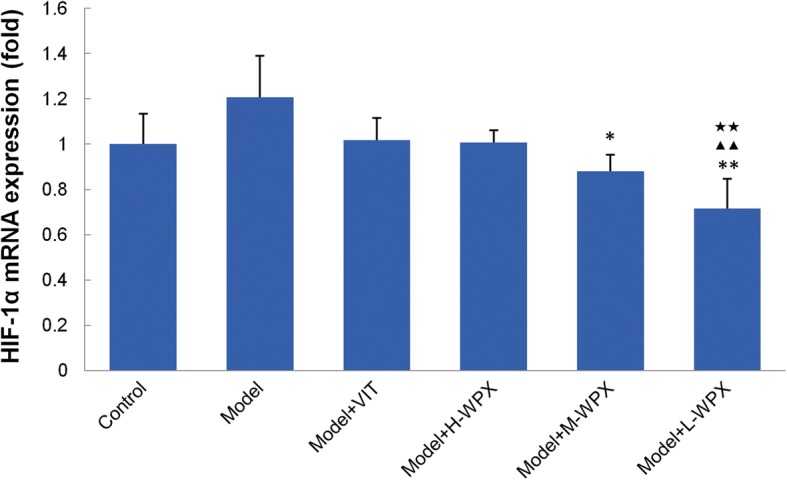
Effect of WPX on HIF-1α mRNA levels in gastric epithelial cells. The results are expressed as mean ± SD (*n* = 9 in each group). Note: ^**^P < 0.01, ^*^P < 0.05, vs Model; ^▲▲^*P* < 0.01, vs VIT; ^★★^*P* < 0.01, vs H-WPX

HIF-1α plays an important role in hypoxic responses and induces the transcription of various genes responsible for tumor angiogenesis, invasion and metastasis. Thus, we texted whether WPX possessed a regulatory ability on hypoxic responses in GPL rats through classic HIF-1α marker detection. Figure 6 clearly showed that HIF-1α mRNA level was elevated in GPL rats when compared with those of controls (although without statistical significance). Compared with model rats, HIF-1α mRNA levels in rats were significantly diminished by medium and low doses of WPX. By comparison, treatment with low dose of WPX led to a marked reduction in HIF-1α mRNA level. The data suggest that WPX, especially at low dose, could efficiently inhibit HIF-1α mRNA expression in GPL rats (Fig. 6).

### Effect of WPX on HIF-1α protein expressions

**Fig. 7:**
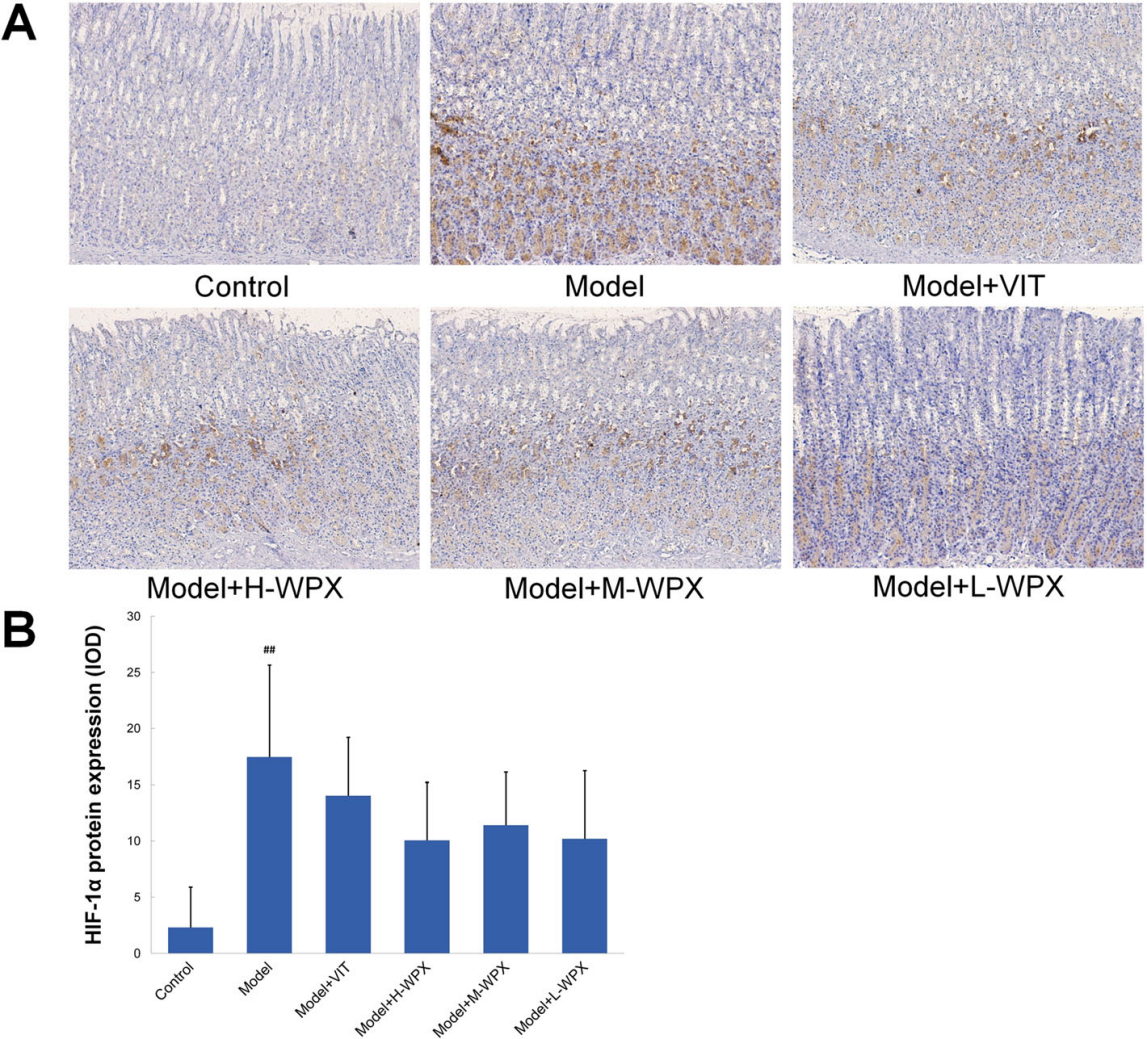
Effect of WPX on HIF-1α protein expressions in gastric epithelium. **a** HIF-1α protein expressions in gastric epithelium from different groups. **b** IOD scores of HIF-1α protein levels. The results are expressed as mean ± SD (*n* = 9 in each group). Note: ^##^*P* < 0.01, vs Control. (IHC, 100×)

We subsequently applied immunohistochemistry to visualize activation of presumptive HIF-1α marker in gastric mucosa. By immunostaining, HIF-1α was sparsely expressed in normal gastric mucosa, whereas HIF-1α positive cells were found relatively abundant in most GPL tissues. Moreover, we found that WPX could visually reduce the number of HIF-1α positive cells in majority of GPL tissues, as shown in Fig. 7a. Semiquantitatively, elevated HIF-1α protein expression was observed in GPL mucosa as compared to normal mucosa. After WPX intervention, we found a clearly downtrend, although without statistical significance, of HIF-1α levels in gastric mucosa. Our results indicated that WPX treatment may produce regulatory effects on hypoxic responses in GPL gastric mucosa (Fig. 7).

### Effect of WPX on VEGF protein expressions

**Fig. 8:**
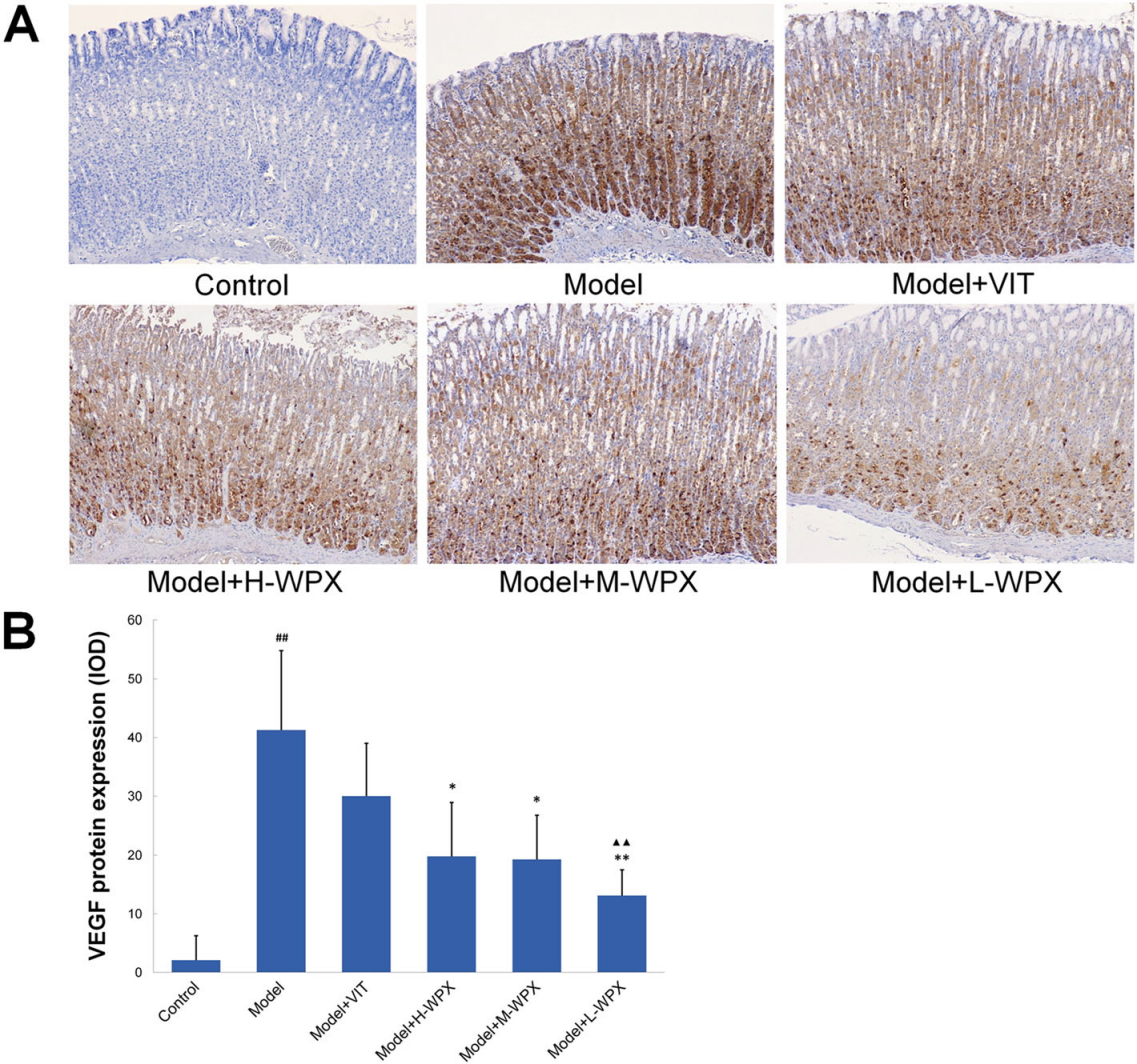
Effect of WPX on VEGF protein expressions in gastric epithelium. **a** VEGF protein expressions in gastric epithelium in various groups. **b** IOD scores of HIF-1α protein levels. The results are expressed as mean ± SD (*n* = 9 in each group). Note: ^##^*P* < 0.01, vs Control; ^**^P < 0.01, ^*^P < 0.05, vs Model; ^▲▲^*P* < 0.01, vs VIT. (IHC, 100×)

VEGF is generally considered as a vital driving force behind the angiogenesis process, thus we next tested whether VEGF inhibition was of relevance for WPX’s anti-angiogenic capacity. As shown in Fig. 8a, normal gastric mucosa did not or barely express VEGF marker, while diffuse and intense cytoplasmic labeling, found in most cases of GPL rats, could be markedly diminished by WPX. Statistically, GPL rats displayed an increased VEGF protein expression compared with negative controls, whereas WPX treatment reduced the over-expression. In addition, we observed a stronger inhibition effect of L-WPX on VEGF over-expression than that of VIT treatment. Intriguingly, we found HIF-1α and VEGF reduction was frequently along with the attenuation of CD34+ angiogenesis in GPL tissues, indicating that HIF-1α and VEGF inhibition may play a beneficial role in WPX-alleviated angiogenesis (Fig. 8).

### Effect of WPX on ERK1, ERK2 and Cyclin D1 mRNA levels

**Fig. 9:**
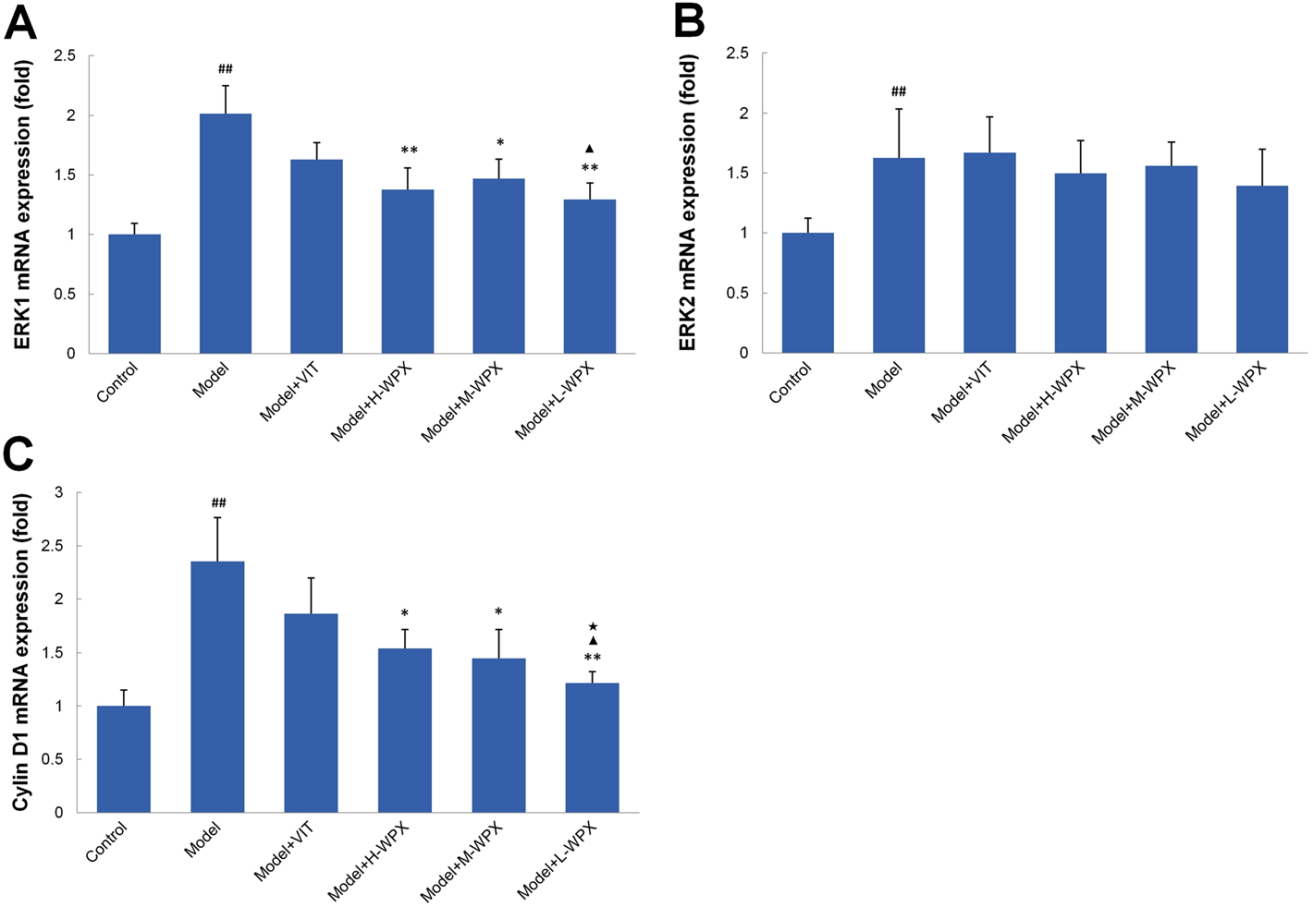
Effect of WPX on the mRNA levels of ERK1, ERK2 and Cyclin D1 in gastric epithelial cells. **a** ERK1 mRNA levels of gastric epithelium in various groups. **b** ERK2 mRNA levels of gastric epithelium in various groups. **c** Cyclin D1 mRNA levels of gastric epithelium in various groups. The results are expressed as mean ± SD (*n* = 9 in each group). Note: ^##^*P* < 0.01, vs Control; ^**^P < 0.01, ^**^P < 0.05, vs Model; ^▲^*P* < 0.05, vs VIT; ^★^*P* < 0.05, vs H-WPX

To identify the possible mechanism underlying the anti-angiogenesis activity of WPX on GPL rats, we examined the key targets ERK1, ERK2 and Cyclin D1, which are closely related to the HIF-1α and EVGF signals. As described above, ERK is a specific effector of VEGF signaling and plays a pro-angiogenic role in sprouting [12], thereby instrumental in the progression of gastric cancer. As shown in Fig. 9, we found the gastric precancerous tissues with dramatically elevated ERK1 mRNA levels, which could be reversed by varying concentrations of WPXs. In addition, low dose WPX was found to be markedly superior to VIT in reducing mucosal ERK1 levels. We also noted an elevated ERK2 mRNA levels in GPL tissues, when in comparison to normal gastric tissues. However, in most cases of WPX-treated rats, upregulated ERK2 mRNA levels remained.

We then analyzed mRNA levels of Cyclin D1, a downstream molecule related to ERK signals. Real-time PCR results revealed that GPL rats exhibited notably elevated Cyclin D1 levels compared with the controls. Importantly, WPX treatment caused a marked drop of Cyclin D1 levels in GPL tissues. Similar to ERK1, the inhibitory activity on Cyclin D1 by WPX was more distinct at low dose. Taken together, our findings implicated that the anti-angiogenesis effect of WPX was achieved partly by suppressing ERK1 and Cyclin D1 activation, and its inhibitory effect was identified to be more potent than that of VIT. However, WPX may have little effect on ERK2 amplification (Fig. 9).

## Discussion

It is well established that gastric carcinogenesis is a complex and multifactorial process, in which accumulation of multiple genetic changes may be implicated. The recognized human model [31] of gastric carcinogenesis comprises the following precancerous steps: superficial gastritis → multifocal atrophic gastritis → intestinal metaplasia → dysplasia. Based on differences in the magnitude of the malignant risk, GPL could be categorized into (1) “non-progressive GPL” (mainly contains S-IM, mild and moderate dysplasia), remaining a comparatively stable status and with a reduced risk of evolving into gastric carcinoma [32, 33], and (2) “progressive GPL” (comprises some cases of C-IM and severe dysplasia), which is more ominous due to a relatively high risk of malignant transformation and requires advisably interval endoscopic and histologic controls [34, 35]. The vast majority of GPL represents a stage within a prolonged process and remains stable, thereby providing an opportunity to block and even reverse the precursors. WPX is a typical Chinese herbal prescription proved clinically effective in treating GPL.

In this project, almost all model rats exhibited GPL pathology, which ranged from moderate IM to severe GED lesion. After WPX administration, IM lesion (including S-IM and C-IM) were markedly regressed in most cases of GPL rats. We also found that WPX could halt and even reverse the majority of mild and moderate dysplasia. However, 4 WPX-treated rats (two M-WPX rats, one H-WPX rat, one L-WPX rat) displayed moderate or severe GED pathology, suggesting that a refractory state to WPX administration might have developed in a certain percentage of “progressive GPL”. Our observations reinforce the view that a few advanced GED and early gastric cancer are partly similar, in terms of cell proliferative activity and cell atypia. Thus, some cases of “progressive GPL” might be difficult to block and reverse.

Microvasculature serves to circulate and transport oxygen and nutrients, which is imperative to various tissues including gastric mucosa. In contrast to the normal microvasculature, cancer-related angiogenesis, which is continually activated and unregulated [36], is a fundamental pathobiological process. It develops a new but malfunctional microvasculature [37], aiming at facilitating oxygen and nutrients supply, and thereby fuels tumor fast-growth. However, the occurrence of angiogenic activity in GPL, and microvessel morphological changes still remain unclear. In this study, we found that CD34+ microvessels were distributed sparsely in normal gastric mucosa, while their number increased significantly in GPL tissue, supporting the hypothesis that early angiogenesis is existed in GPL rats. Interestingly, in more advanced lesions, gastric mucosa frequently exhibited a higher CD34+ microvessel count. We found more GEDs with a higher number of microvessels than IMs, and more severe GEDs than mild or moderate GEDs. Notably, the most numerous CD34+ microvessels were detected in two model rats with severe GED. The above mentioned phenomena may reveal that gastric precancerosis were frequently heterogeneous in angiogenic behavior, and that a significant higher angiogenic state may imply an increased potential biological attitude towards malignancy, which is in agreement with a previous study [38]. Micromorphologically, the microvessel ultrastructural alterations found in GPL tissue were mainly characterized by dilated vascular lumen, clearly thickened and rough basal lamina, and also by conglobated, degenerated endothelial cell. These characteristics suggest that microvascular abnormalities and hypoxia vasodilation often co-existed in GPL rats. This subsequently induces hypoxia stress together with the activation of hypoxia-inducible factors [39], which stimulate secretion of VEGF and angiogenesis. Hence, angiogenesis may be an adaptive pathobiological response, often accompanying with microvascular abnormalities, triggered by microenvironmental hypoxia in gastric mucosa, aiming at restoring O_2_ delivery to hypoxic regions. We speculated that chronic inflammation is a prominent inducer, which could result in microvascular injury [40] and also render gastric tissues more hypoxic [41], and therefore drive angiogenesis [42]. (inflammatory infiltration and altered expressions of inflammatory cytokines TNF-α and IL-4 were observed in GPL rats revealed by our previous study [43]). Nonetheless, these were just our preliminary findings, detailed information concerning microcirculation blood flow, inflammation-induced hypoxia and angiogenesis in GPL tissues became our next focus.

Interestingly, WPX administration could rescue microvascular abnormalities and attenuate early angiogenesis in most of the specimens with a concomitant regression of IM and GED lesions. These findings suggested that WPX might possess multi-functions in blocking the GPL aggravation, not only by its anti-angiogenesis ability revealed by a marked drop of MVD level, but also, probably the most important, by ameliorating the microvascular abnormalities and the subsequent microcirculatory dysfunction. HIF-1α and VEGF have been implicated as classic factors controlling multiple proangiogenic processes hijacked by hypoxic tumors, aimed at normalizing blood flow [13]. In this study, we noted that early angiogenesis observed in GPL tissue is paralleled by HIF-1α and VEGF activation. More importantly, WPX could suppress the hypoxia-triggered accumulation of HIF-1α and the VEGF activation, this result supports the hypothesis that HIF-1α and VEGF inhibition plays a beneficial role in WPX-alleviated angiogenesis. While HIF-1α mRNA levels were elevated in GPL tissues, and the number of HIF-1α positive cells was visually reduced after WPX treatment, we archived no statistically significant differences. Given the hypoxia was a heterogeneous concept with uneven oxygen tensions in localized regions [44], spatial maldistribution of HIF-1α activation in GPL tissues may factor in these non-significant differences. Besides, sample size limitation may be another possible contributor.

Angiogenesis is a complex multistep process regulated by compounding factors. It is proposed that ERK/Cyclin D1 could act as specific effectors of VEGF signaling to elicit excessive angiogenesis behavior, and thus facilitated cell proliferation, suggesting a crucial role of the molecules in the initiation and progression of gastric cancer. However, what role the molecules may play in GPL is less clear. In this study, as expected, up-regulated mRNA expressions of ERK1, ERK2 and Cyclin D1 were observed in gastric mucosa from GPL rats as compared to normal mucosa. Our results bring a crucial contribution by evidencing that hyper-angiogenesis observed in GPL is driven, in part, through aberrant activation of ERK-related molecules, suggesting their involvement in the malignant transformation. We were also curious as to whether inhibition of ERK signals is involved in the underlying mechanisms of WPX-mediated attenuation of angiogenesis. Interestingly, after WPX intervention, a decrease in ERK1levels was frequently concurrent with the reduction of CD34+ microvessels. Hence, this ERK mitigating effect might be contributed to the anti-angiogenic activity of WPX against precursor lesions. In addition, no significant decrease in ERK2 levels was observed in WPX-treated rats, which might be due to sample size limitation, or to the speculation that ERK2 might not be the potential therapeutic target for GPL with WPX.

Previously, we have found that H-WPX and M-WPX were more superior to L-WPX in ameliorating gastric precancerosis [45], as well as suppressing cell proliferation and promoting apoptosis [46]. In this study, conversely, L-WPX showed a relatively better anti-GPL activity by attenuating early angiogenesis, and by regulating ERK-related molecules as compared to those of H-WPX and M-WPX. We speculate that different treatment duration (10 weeks in the present study, 4 weeks in the previous studies) might be responsible for the inconsistency. It remains possible that WPX, at low dose, might be more efficient for long-term intervention of GPL when compared with that at high and medium doses. However, due to the small sample size, the speculation need to be further validated in larger scale studies. Our HPLC analysis revealed that Calycosin-7-glucoside, ginsenoside-Rg1, ginsenoside-Rvb1, astragaloside IV, as well as atractylenolide III, II and I might be the potential anti-angiogenic candidates of WPX. Ginsenoside Rg1 has been reported to suppress the vascular neointimal hyperplasia by inhibiting on ERK2 signaling [47]. Ginsenoside Rb1 displayed anti-angiogenesis through suppressing the formation of endothelial tube-like structures [48]. Besides, atractylenolide I displayed a potent inhibitory effect on angiogenesis driven by chronic inflammation in vivo and vitro [49]. Although the aforementioned reports likely support the anti-angiogenesis capacity of WPX, there are some caveats. For instance, there have been conflicting reports regarding the pro-angiogenic role [50, 51] or anti-angiogenic role [52] of astragaloside IV. Thus, much more work remains to be addressed in order to fully exploit anti-angiogenic potential of the compounds in MNNG-induced GPL rats and other GPL models in the future.

## Conclusion

In summary, WPX could attenuate the angiogenic response and temper microvascular abnormalities in GPL rats. The anti-angiogenesis property might be related to inhibition on the angiogenesis-associated markers HIF-1α and VEGF, and on the ERK1/Cylin D1 aberrant activation. Additional files 1, 2, 3, 4, 5, 6 and 7.

## Additional files

Additional file 1: CD34 (IHC-IOD). Raw data for Fig. 4. (XLS 23 kb)

Additional file 2: HIF-1alpha (PCR). Raw data for Fig. 6. (XLS 27 kb)

Additional file 3: HIF-1alpha (IHC-IOD). Raw data for Fig. 7. (XLS 21 kb)

Additional file 4: VEGF (IHC-IOD). Raw data for Fig. 8. (XLS 23 kb)

Additional file 5: ERK1 (PCR). Raw data for Fig. 9. (XLS 23 kb)

Additional file 6: ERK2 (PCR). Raw data for Fig. 9. (XLS 23 kb)

Additional file 7: Cyclin-D1(PCR). Raw data for Fig. 9. (XLS 27 kb)

## Abbreviations

ANOVA: Analysis of variance
ERK: Extracellular signal-regulated kinase
GED: Gastric epithelial dysplasia
GPL: Gastric precancerous lesions
H&E: Hematoxylin and eosin
HID-AB-PAS: High-iron diamine- alcian blue-periodic acid schiff
HIF-1α: Hypoxia inducible factor-1α
HPLC: High performance liquid chromatography
IM: Intestinal metaplasia
IOD: Integrated optical density.
MNNG: N-methyl-N′-nitro-N-nitrosoguanidine
MVD: Microvessel density
RT-qPCR: Quantitative real-time reverse transcription-polymerase chain reaction
TEM: Transmission electron microscope
TNM: Tumor node metastasis
VEGF: Vascular endothelial growth factor
VIT: Vitacoenzyme
WPX: Weipixiao
